# Encapsulation of Menthol in Bimodal Mesoporous Silica via Normal-Temperature and Alcohol-Thermal Loading Methods for Achieving Sustained Releasing Performances

**DOI:** 10.3390/nano16090545

**Published:** 2026-04-29

**Authors:** Yuhua Bi, Tiejun Ma, Andong Wang, Fei Liu, Ruohan Xu, Tallat Munir, Jihong Sun, Wenliang Fu, Donggang Xu

**Affiliations:** 1 Beijing Key Laboratory for Green Catalysis and Separation, Institute of Matter Science, Beijing University of Technology, Beijing 100124, China; biyuhua@emails.bjut.edu.cn (Y.B.); matiejun@bjut.edu.cn (T.M.); wangad@bjut.edu.cn (A.W.); liufei@emails.bjut.edu.cn (F.L.); xuruohan@emails.bjut.edu.cn (R.X.); ch023tm@kust.edu.pk (T.M.); 2 Beijing Institute of Basic Medical Sciences, Beijing 100850, China; xudg@bmi.ac.cn

**Keywords:** bimodal mesoporous silica, menthol encapsulation, alcohol-thermal loading, sustained release, mesoporous adsorption, molecular diffusion

## Abstract

**Background**: Menthol is a naturally occurring volatile terpene alcohol, widely used in food, pharmaceutical, and tobacco products; however, its high volatility leads to significant flavor loss during storage and handling. **Methods**: Herein, bimodal mesoporous silica materials (BMMs) were employed as carriers to encapsulate menthol, the loading and release behaviors were systematically compared using normal-temperature and alcohol-thermal loading methods. **Results**: Comprehensive characterizations (XRD and SAXS patterns, FT-IR spectra, SEM images, and N_2_-sorption isotherms) confirmed that menthol incorporation did not disrupt the hierarchical mesoporous channels of BMMs. The alcohol-thermal loading method achieved a superior menthol loading capacity of 87%, significantly outperforming the normal-temperature loading (58%). Release performances revealed a transition in the dominant release mechanism, from diffusion-controlled behavior at low loading levels to concentration gradient-driven desorption at high loadings. Molecular dynamics simulations further demonstrated that alcohol-thermal loading enabled faster molecular diffusion and a more uniform distribution of menthol within the mesopores due to weaker interfacial interactions, whereas normal-temperature loading induced localized multilayer adsorption, resulting in mesopore blockage and hindered diffusion. In addition, long-term atmospheric release tests assessed sustained menthol retention over 30 days. **Conclusions**: Overall, this work establishes alcohol-thermal loading as an effective approach for regulating adsorption and release in mesoporous carriers, providing a foundation for developing volatile compound encapsulation strategies.

## 1. Introduction

Menthol, as one of the most important additives in food, daily necessities, medicine, and tobacco [1,2], usually provides a unique sense of coolness with antibacterial, anti-inflammatory, and anti-cancer effects [3,4], due to its naturally occurring volatile and cyclic terpene alcohol with a characteristic minty smell and flavor [5]. Meanwhile, the median lethal dose (LD50) of menthol in rats and mice was approximately 4000 mg/kg or higher, confirming its high biosafety [6]. Fragrances such as menthol possess the ability to modulate the central nervous system, and aromatherapy has been widely utilized to enhance cognitive function [7]. The scent released by menthol is captured and perceived by the human olfactory system. These signals are subsequently transmitted to the brain, thereby regulating cognitive function [8]. For instance, the additive menthol in cigarettes can release a refreshing taste [9], which is beneficial to stimulate the central nervous system and thereby promote a refreshing and revitalizing effect. However, the high volatility of menthol significantly limits its stability during storage and processing, resulting in rapid evaporation and substantial flavor loss [10,11]. To mitigate these limitations, various encapsulation strategies have been explored to improve menthol retention and regulate its release behavior. Among them, various carriers, such as β-cyclodextrin, starch, chitosan, and mesoporous silica, have been extensively reported for menthol encapsulation [12,13,14,15]. In particular, Shi et al. [12] prepared high-amylose corn starch–menthol inclusion complexes, showing an effective encapsulating capacity of around 6% and a controlled release rate of 0.17 g·L^−1^. Wang et al. [13] investigated the effects of substitution degree in octenyl succinic anhydride-modified starch and demonstrated that menthol release from spray-dried microcapsules was negatively correlated with substitution degree during simulated oral processing. Despite these advances, conventional polymeric carriers exhibit low loading capacity, poor mechanical strength, and rigid network structure, restricting their applicability in efficient loading and controlled release systems. Consequently, the development of alternative carrier materials capable of overcoming these limitations remains highly desirable.

Recently, Hu et al. [14,15] reported the used of a β-cyclodextrin metal–organic framework (β-CD-MOF) for menthol encapsulation, achieving a maximum loading capacity of 27.1% and an encapsulation efficiency of 30.6% at the optimal combination of conditions, while menthol retention remained limited to 13.1% under elevated temperature conditions. To further improve storage stability and release performance, Santos et al. [16] developed menthol–xylitol microcapsules via a double-emulsion and complex coacervation approach, which enhanced cooling sensation and enabled controlled release during chewing. Peng et al. [17] prepared α-lactalbumin/gum arabic microparticles using spray-freeze drying, achieving encapsulation efficiencies of 78%, although approximately 50% of the loaded menthol was released within one day. These studies indicate that although existing encapsulation systems can partially improve menthol stability, challenges related to long-term retention and controlled diffusion persist.

Since the discovery of the MCM-41 family in 1990 [18], many researchers focused on the application of the mesoporous silicas as potential drug delivery carriers owing to their nontoxicity, good biocompatibility, high specific surface area, large pore volume, and tunable pore structures [19,20]. Typical mesoporous silicas such as MCM-41 and SBA-15, characterized by one-dimensional pore channels, have been successfully employed for the encapsulation of pharmaceutical compounds including ibuprofen [21], vancomycin [22], and aspirin [23]. Dong et al. [24], reported that menthol-loaded MCM-41 exhibited a loading capacity of approximately 16% and a release duration exceeding 30 days under ambient conditions, outperforming three-dimensional MCM-48 and larger-pore SBA-15. Zhang et al. [25] further demonstrated a thermally triggered menthol release system based on porous silica coated with cellulose acetate, in which the menthol loading increased from 248 mg/g at 373 K to 400 mg/g at 413 K. Nevertheless, the influence of hierarchical pore architectures, spanning micro- to mesoporous length scales, on menthol storage capacity and release behavior has not been thoroughly investigated, and the underlying adsorption–diffusion mechanisms within confined mesoporous environments remain insufficiently understood.

Recently, several groups [26,27] elucidated the mesoporous carriers having interconnecting three-dimensional pore networks that not only increase the storage capacity of the used drugs, but also facilitate molecular diffusion. Obviously, these demonstrations suggest that the rational design of hierarchical mesoporous structures represents a promising strategy for achieving optimized delivery performance. Building on this concept, our group previously reported functionalized bimodal mesoporous silica materials (BMMs) as efficient drug carriers with high encapsulation efficiency and loading capacity [28,29,30,31,32], attributed to their high surface area (700–1000 m^2^/g), higher pore volume (3.5 cm^3^/g) and hierarchical pore architecture. In particular, the coexistence of uniform small mesopores (~2.5 nm) and broadened intraparticle mesopores (10–50 nm) provides enhanced accessibility and diffusion pathways for guest molecules. Representative studies have shown that amino-functionalized BMMs achieve aspirin loading capacities as high as 54% with sustained release in simulated body fluids [33], while core–shell PMAA@BMMs composites exhibit pH-responsive behavior and improved ibuprofen release under neutral conditions [34]. Particularly, our recently reported results regarding cell (HepG-2 and HeLa) viability and hemolysis rate experiments preliminarily provided obvious evidence for excellent blood biocompatibility and low cytotoxicity in BMMs-based delivery systems [35,36].

Despite these advances, the encapsulation of volatile small molecules such as menthol within hierarchical mesoporous systems remains largely unexplored, particularly with respect to how different loading pathways govern adsorption diffusion behavior and pore utilization. Herein, the bimodal mesoporous silica materials (BMMs) were used as a carrier to achieve high loading capacity for menthol and thereafter sustained release performance. Menthol was loaded into the mesoporous channels of BMMs via normal-temperature and alcohol-thermal loading methods, respectively. The novelties of the present work demonstrated the detailed comparisons between these loading strategies to elucidate their distinct effects on loading efficiency, diffusion kinetics, and adsorption properties. The structural features and physicochemical properties were characterized using various characterizations. Particularly, the SAXS patterns provided the quantitative insight into the fractal structural evolution during loadings. Furthermore, the long-term sustained release behaviors were evaluated over 30 days under atmospheric conditions. The diffusion mechanism of the menthol in the mesopore channels of the BMMs matrix was elucidated by combining experimental results with kinetic and molecular dynamics simulations, in which the adsorption kinetics (the pseudo-first-order (PFO), pseudo-second-order (PSO), and intraparticle diffusion (IPD) models), adsorption isotherms (Langmuir, Freundlich, Temkin, and Dubinin–Radushkevich (D–R) models), releasing kinetics (first-order, Higuchi, and Korsmeyer–Peppas (K–P) models), and thermodynamic parameters (Gibbs free energy change (Δ*G*), enthalpy change (Δ*H*), and entropy change (Δ*S*)), were employed to elucidate the menthol release behaviors. Meanwhile, molecular dynamics (MD) simulations were used to reveal diffusion coefficients, radial distribution function (RDF), and the molecular configuration of menthol under both normal-temperature and alcohol-thermal loading conditions.

## 2. Materials and Methods

### 2.1. Chemicals

Ammonia (AR, 25–27%) and anhydrous ethanol (AR) were obtained from Fuchen (Tianjin) Chemical Reagent Co., Ltd., (Tianjin, China). Menthol (99%) was provided by Ji’an Zhongxiang natural plant Co., Ltd. (Ji’an, China). Cetyltrimethylammonium bromide (CTAB, 99%), deuterated chloroform (99.8%), and tetraethyl orthosilicate (TEOS, 98%) were purchased from Bailingwei Technology (Beijing) Co., Ltd. (Beijing, China). All reagents were used as received without purification.

### 2.2. Experimental Procedure

#### 2.2.1. Preparation of BMMs

BMMs were synthesized following a reported method with minor modifications [37]. In details, 10.44 g CTAB was dissolved in 416 mL of distilled water at 40 °C under continuous magnetic stirring until a clear solution was obtained. Subsequently, 32 mL TEOS was added dropwise to the solution, followed by the rapid addition of 9.6 mL of ammonia solution (25–27%). The mixture immediately turned into a white gel. The resulting product was collected by filtration, thoroughly washed with distilled water, and dried at 80 °C for 5 h. The dried solid was then calcined in a furnace by heating from room temperature to 550 °C at a rate of 5 °C min^−1^ and maintained at this temperature for 5 h. The final product was denoted as BMMs.

#### 2.2.2. Menthol Loading

Menthol loading process was carried out by immersion of BMMs (0.2 g) in menthol ethanol solution (100 mL) with a designated concentration (5–30 mg/mL) at a fixed temperature for a specified time. After filtering, the resultant solid was denoted as BL-x-y-z (BL stands for the menthol-loaded BMMs matrixes prepared via normal-temperature loading method, while, x, y and z represent a menthol concentration (mg/mL) in ethanol, loading temperature (°C), and loading time (min), respectively). The remaining solution was collected and diluted for subsequent gas chromatography tests.

For alcohol-thermal loading, menthol and BMMs were mixed at a predetermined ratio and placed in a sealed pressure vessel, followed by continuous heating. After cooling to room temperature, the solids and liquids were separated via centrifugation. The obtained solutions were collected and diluted for subsequent gas chromatography tests. The solids were washed with 10 mL absolute ethanol to remove non-loaded menthol (only once to minimize the loss of mesopore-loaded menthol), and then dried at 65 °C over 3–5 h. The resulting samples were denoted as BG-x-y-z, where BG indicates alcohol-thermal loading and x, y, and z represent menthol concentration (mg·mL^−1^), loading temperature (°C), and loading time (min), respectively.

The loading capacity of BG samples was systematically regulated by varying the loading temperature (60, 80, 100, 120, 140 °C), loading time (10, 30, 60, 120, 240 and 360 min), and menthol concentration (280, 360, 420, 470, 510, 550, 570, 600, 620, 640, 660, 680 mg/mL). Table S1 in the Electronic Supporting Information (ESI) section summarizes the synthesis parameters of the menthol-loaded BMMs prepared via normal-temperature and alcohol-thermal loading methods. Meanwhile, Scheme S1 in the ESI section illustrates the preparation process for loading and releasing menthol.

The loading capacity was calculated using Equation (1):

(1) $$\mathrm{Loading}\;\mathrm{Capacity}\;(\%)\;=\;\frac{M_{0}\;-{\;M}_{1}}{M_{2\;}+{\;M}_{0}\;-\;M_{1}}\;\times \;100\%$$

where *M*_0_ (g) and *M*_1_ (g) are the initial and remaining menthol masses in solution, respectively, and *M*_2_ is the mass of BMMs (g).

#### 2.2.3. Menthol-Releasing Behavior

Menthol release experiments were conducted by placing 0.05 g of BL or BG samples into a dialysis bag and dispersing it in 50 mL of anhydrous ethanol. The system was maintained in a constant-temperature air oscillator. At predetermined time intervals, 2 mL of the release medium was withdrawn and replaced with an equal volume of fresh ethanol to maintain a constant total volume. Menthol concentration was quantified by gas chromatography (GC), and the recovered solids were denoted as BL-x-y-z-SF or BG-x-y-z-SF.

The cumulative release amount was calculated using Equation (2):

(2) $$\mathrm{Cumulative}\;\mathrm{Release}\;\mathrm{Amount}\;(\mathrm{mg}/\mathrm{mL})\;=\;C_{t}\;+\;\frac{V_{1}}{V_{2}}\sum_{0}^{t-1}C_{t}$$

where *C_t_* (mg/mL) is the menthol concentration at time *t*, *V*_1_ (mL) is the measured volume of the released medium, *V*_2_ (mL) is the total volume of the release medium.

For open-system release tests, 5 g of pure menthol and BG-640-80-60 were uniformly spread onto circular trays (diameter: 30 cm; area: 706.5 cm^2^), covered with a matching sieve, and placed indoors. The mass loss was recorded at specific intervals (1, 2, 4, 7, 10, 14, 21, and 30 days) to determine menthol volatilization.

The cumulative release percentage was calculated using Equation (3):

(3) $$\mathrm{Cumulative}\;\mathrm{Release}\;\mathrm{Percent}\;(\%)\;=\;\frac{{M_{1}\;-\;M}_{t}}{M_{0}}\times 100\%$$

where *M_t_* (g) is the sample mass at time *t*, *M*_1_ (g) is the initial total sample mass, and *M*_0_ (g) is the initial menthol mass.

### 2.3. Characterizations

The XRD patterns were collected using a D6 Advance diffractometer (Beijing Pu Analysis General Instrument Co., Ltd., Beijing, China) with Cu-Kα radiation (λ = 0.154056 nm, 36 kV, 20 mA) over a 2*θ* range of 1–10° at a scanning rate of 1° min^−1^. Textural characteristics of the samples were evaluated through nitrogen adsorption–desorption measurements at 77 K using a JWGB JW-BK300 surface area analyzer (Beijing Jingwei Gaobo Instrument Co., Ltd., Beijing, China). Samples were degassed under vacuum at 298 K for 1 h prior to analysis. The specific surface area was determined by the Brunauer–Emmett–Teller (BET) method, pore size distribution was derived from the BJH model, and total pore volume was estimated at *P*/*P*_0_ = 0.99. TG measurement was performed on a PerkinElmer STA-8000 instrument (PerkinElmer, Waltham, MA, USA) under a nitrogen atmosphere (flow rate: 20 mL·min^−1^), with the temperature ramped from 30 to 900 °C at a rate of 10 °C·min^−1^. Morphological features were examined by field-emission scanning electron microscopy (FE-SEM, Hitachi S-4800, Tokyo, Japan) at an accelerating voltage of 15.0 kV. The mesoporous structures of related samples were observed using a transmission electron microscope (TEM, JEOL JEM-2100F, Tokyo, Japan). FT-IR spectra were acquired on a PerkinElmer Spectrum 100 spectrometer (PerkinElmer, USA) with a spectral resolution of 4 cm^−1^, accumulating 16 scans per sample using the KBr pellet method. ^1^H NMR spectra were recorded on an ASCETM 400 (AVANCE HD III) spectrometer (Bruker, Ettlingen, Germany) operating at a resonance frequency of 400 MHz, using a 5 mm NMR tube and deuterated chloroform as the solvent. Before determinations, 150 mg of BG-640-80-60 was dispersed in 1 mL of deuterated chloroform, and then performed via ultrasonic concussion for half an hour. After centrifuging, the obtained menthol-containing solution was transferred into NMR sample tubes for ^1^H NMR analysis, while 70 mg of pure menthol was dissolved in 1 mL of the deuterated chloroform for ^1^H NMR determination.

The SAXS experiments were performed at the 1W2A beamline of the Beijing Synchrotron Radiation Facility (BSRF) with an X-ray wavelength of 0.154 nm. The scattering vector *q* ranged from 0.08 to 3.05 nm^−1^. Data processing was conducted using Fit2D and S programs to obtain mass fractal dimensions (*D_m_*) and surface fractal dimension (*D_s_*) values. GC analysis was performed using a GC-2010Pro system (Shimadzu, Kyoto, Japan) equipped with a DB-WAX capillary column (Agilent, Santa Clara, CA, USA) and FID detector.

### 2.4. Adsorption Kinetics

Adsorption kinetics data were evaluated using PFO [38], PSO [39], and IPD [40] models, expressed by Equations (4)–(6):

(4) $$Q_{t}=q_{e}(1-e^{-K_{1}t})$$

(5) $$Q_{t}=(K_{2}q_{e}^{2}t)/(1+K_{2}q_{e}t)$$

*Q_t_* = *K_i_t*_1_/2 + *C_i_* (6)

where *Q_t_* (mg·g^−1^) and *q_e_* (mg·g^−1^) are the amounts of drug sorbed at time *t* and equilibrium, respectively. *K*_1_ (min^−1^), *K*_2_ (g·mg^−1^·min^−1^), and *K_i_* (mg·g^−1^·min^−1/2^) denote the rate constants of PFO, PSO, and IPD models, and *C_i_* (mg·g^−1^) is the intercept.

### 2.5. Adsorption Isotherms

Equilibrium adsorption data were fitted using the Langmuir [41], Freundlich [42], Temkin [43], and D–R [44] models, expressed by Equations (7)–(10):

*q_e_* = *q_m_K_L_C_e_*/(1 + *K_L_C_e_*) (7)

(8) $$q_{e}=K_{F}C_{e}^{1/n}$$

*q_e_* = *RT*/*b ln*(*AC_e_*) (9)

(10) $$q_{e}=(q_{\mathrm{DR}})e^{-K_{\mathrm{DR}}\varepsilon^{2}}$$

where *q_e_* is the equilibrium adsorption capacity, *C_e_* is the equilibrium concentrations, *q_m_* is the maximum adsorption capacity. *K_L_*, *K_F_*, and *n* are the Langmuir and Freundlich constants and heterogeneity factor, respectively. *R* is the gas constant (8.314 J·mol^−1^·K^−1^), *T* is the absolute temperature (K), *b* and *A* are Temkin constants, *q_DR_* is the D–R constant, and *ε* is the Polanyi potential.

### 2.6. Adsorption Thermodynamic

Thermodynamic parameters, including Δ*G*, Δ*H*, and Δ*S*, were calculated using Equations (11)–(13) [45].

(11) ΔG=−RTlnK

(12) ΔG=ΔH−TΔS

(13) $$\mathrm{lnK}=\frac{\Delta S}{R}-\frac{\Delta H}{\mathrm{RT}}$$

where *K* = *q_e_*/*C_e_*, *R* is the gas constant, and *T* (K) is the absolute temperature.

### 2.7. Release Kinetics Behaviors

Menthol release behavior was evaluated using the first-order [46], Higuchi [47], and K–P models [48], as shown in Equations (14)–(16):

(14) $$M_{t}/M_{\infty }=1-e^{-K_{1}t}$$

*M_t_*/*M_∞_* = *K_H_ t*^1/2^ (15)

*M_t_*/*M_∞_* = *K_KP_ t^n^* (16)

where *M_t_* and *M_∞_* are the released amounts at time *t* and equilibrium, respectively. *K*_1_, *K_H_*, and *K_KP_* are the corresponding rate constants and *n* indicates the release mechanism.

### 2.8. MD Simulation

MD simulations were performed using Materials Studio to examine menthol diffusion and interfacial interactions within BMMs. The BMMs model was constructed based on the MCM-41 framework [49]. The initial structure was created by expanding the built-in amorphous SiO_2_ unit cell (SiO_2_ 21A 3d) in MS. Based on experimental pore data, the worm-like channels of BMMs were simplified into a single-channel model, with a channel diameter of 2.6 nm and a sinusoidal pore shape (A = 0.115 nm). The unit cell dimensions were set to 4.201 × 6.301 × 8.402 nm, as shown in Figure S1A of the ESI section. Using the Locate function in the Sorption module of MS, menthol and ethanol molecules were inserted into the model. Menthol and ethanol molecules were introduced according to experimental loading conditions: 10 menthol molecules/50 ethanol molecules at 298 K for normal-temperature loading and 30 menthol molecules/30 ethanol molecules at 358 K for alcohol-thermal loading. The menthol-loaded BMMs model via the normal-temperature loading method and alcohol-thermal loading method is shown in Figure S1B,C.

The MD simulations were performed using the Forcite module to investigate the diffusion behavior of menthol molecules within BMMs. The calculations employed the COMPASS-III force field with fine accuracy settings. The system pressure was maintained at 1 MPa, with electrostatic interactions calculated by the Ewald method and van der Waals interactions treated by an atom-based summation scheme. The constructed pore model initially underwent three cycles of annealing. The resulting structure was then equilibrated under the NPT ensemble for 50 ps. Subsequently, the system was switched to the NVT ensemble and further equilibrated for 100 ps. After equilibration, production runs were performed under the NVE ensemble for 1000 ps.

Based on Density Functional Theory (DFT), the geometry optimization of the free-state menthol molecular model was performed using the DMol^3^ module. The exchange-correlation functional was selected as the Perdew–Burke–Ernzerhof (PBE) functional within the generalized gradient approximation (GGA). Calculations were carried out using a double numerical plus polarization (DNP) basis set. The Hexapole method was employed for self-consistent field (SCF) iteration, ensuring reliable convergence and numerical stability of the algorithm. The energy convergence criterion was set to 1.0 × 10^−5^ Ha, and the geometry optimization convergence thresholds were set as follows: maximum force not exceeding 0.02 Ha/nm, and maximum displacement not exceeding 0.0005 nm.

The mean squared displacement (MSD) of the menthol molecules was computed by analyzing the simulation trajectories. Diffusion coefficients were obtained from mean-squared displacement analysis using the Einstein relation, and menthol–BMMs interactions were evaluated via radial distribution functions.

The self-diffusion coefficient (*D*) can be calculated from MSD using Equation (17) [50]:

(17) $$D\;=\;\frac{1}{6}\underset{t\to \infty }{\lim}\frac{d}{\mathrm{dt}}{\langle |r_{i}(t)\;-\;r_{i}(0)|}^{2}\rangle$$

where *r_i_*(*t*) is the center-of-mass position vector of molecule *i* at time *t*.

The RDF is defined as the relative probability of finding an atom at a distance *r* from a reference atom. Its mathematical expression is given in Equation (18) [51]:

(18) $$g(r)\;=\;\frac{1}{4\pi r^{2}\rho }\frac{\mathrm{dN}(r)}{\mathrm{dr}}$$

where *dN* is the number of atoms occurring in the range from *r* to *r* + ∆*r*, and *ρ* denotes the average density of the system.

## 3. Results

### 3.1. Characterization of Menthol-Loaded and -Released BMMs

**Figure 1:**
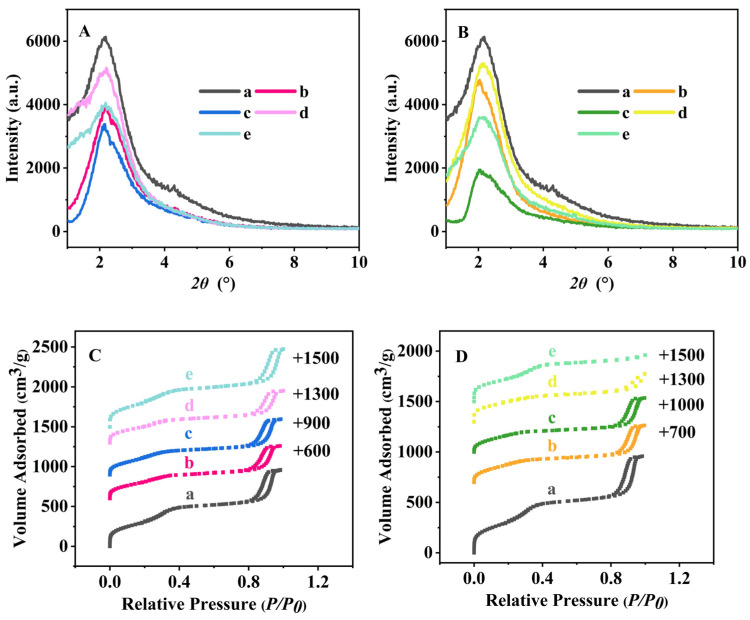
XRD patterns (**A**) and N_2_ adsorption–desorption isotherms (**C**) of samples prepared via normal-temperature loading method. BMMs (a), BL-5-25-720 (b), BL-25-25-720 (c), BL-5-25-720-SF (d), and BL-25-25-720-SF (e). XRD patterns (**B**) and N_2_ adsorption–desorption isotherms (**D**) of samples prepared via alcohol-thermal loading method. BMMs (a), BG-640-80-10 (b), BG-640-80-60 (c), BG-640-80-10-SF (d), and BG-640-80-60-SF (e).

Figure 1A,B presents the XRD patterns of before and after menthol-released BMMs. As can be seen, the XRD patterns of BMMs (Figure 1A (a)) exhibited a distinct (100) reflection at 2*θ* = 2.20°, indicating the existence of ordered mesopore structures [52]. After menthol loading, either with various concentrations or different times, the samples prepared via normal-temperature and alcohol-thermal loading methods both showed a distinct diffraction peak at around 2*θ* of around 2.16°, suggesting that the mesopore structures in the menthol-loaded BMMs remained intact.

However, as the menthol-loaded concentrations increased from 5 to 25 mg·mL^−1^, the characteristic peak (100) of BL-5-25-720 (Figure 1A (b)), BL-25-25-720 (Figure 1A (c)) prepared via normal-temperature loading not only presented the gradually decreased tendencies in intensity, but also shifted to the small 2*θ* scale from 2.20° for BMMs (Figure 1A (a)) to 2.16° for BL-5-25-720 (Figure 1A (b)) and BL-25-25-720 (Figure 1A (c)). Correspondingly their *d* space values increased from 4.01 nm for BMMs (Figure 1A (a)) to 4.09 nm for BL-5-25-720 (Figure 1A (b)) and BL-25-25-720 (Figure 1A (c)). Similarly, the menthol-loaded samples prepared via the alcohol-thermal loading method also showed a shift towards the small 2*θ* regions with increasing menthol-loaded time, showing 2.20° for BMMs (Figure 1B (a)) to 2.04° for BG-640-80-10 (Figure 1B (b)), and 2.02° for BG-640-80-60 (Figure 1B (c)), respectively. Correspondingly their *d* space values increased from 4.01 nm for BMMs to 4.33 nm for BG-640-80-10 (Figure 1B (b)), and 4.37 nm for BG-640-80-60 (Figure 1B (c)), respectively. Obviously, these observations suggested the successful loading of menthol into the mesoporous channels of BMMs. However, the diffraction peak (100) intensity of the menthol-loaded samples prepared via the alcohol-thermal loading method diminished considerably, implying that the successful loading of menthol into the mesoporous channels of BMMs caused the decline in the degree of mesoporous ordering of BMMs.

On the other hand, the XRD patterns of the menthol-released samples, such as BL-5-25-720-SF (Figure 1A (d)), BL-25-25-720-SF (Figure 1A (e)), BG-640-80-10-SF (Figure 1B (d)), and BG-640-80-60-SF (Figure 1B (e)), still exhibited obvious diffraction peaks at about 2*θ* = 2.18°, indicating the maintenance of the mesoporous structures in the menthol-released BMMs. Interestingly, the characteristic peaks of these menthol-released samples shifted to the small 2*θ* scales, as compared with that of BMMs, implying that a fraction of menthol may still reside within the mesoporous channels of the released BMMs.

N_2_ adsorption–desorption isotherms and corresponding pore size distributions of related samples are shown in Figure 1C,D and Figure S2. As can be seen, all samples exhibited type IV adsorption–desorption isotherms with H1-type hysteresis loops, indicating mesoporosity. Among them, two distinct hysteresis loops appeared at pressure ranges of 0.2–0.4 and 0.8–0.98, corresponding to capillary condensation in the primary mesopores and intraparticle mesopores, respectively. The specific surface area and the pore volume of the BMMs (Figure 1C (a)) were determined to be 1254 m^2^/g and 1.62 cm^3^/g, respectively. Its pore size distribution curves, as shown in Figure S2, exhibited the appearance of the bimodal mesopore structures with a narrow small pore distribution around 2.58 nm and a large pore distribution around 16.69 nm, respectively.

After normal-temperature loading, both the BET surface area and pore volume decreased markedly to 946 m^2^/g and 1.20 cm^3^/g for BL-25-25-720 (Figure 1C (c)). Correspondingly, the primary pore size also decreased from 2.58 nm (BMMs) to 2.40 nm (BL-5-25-720, Figure 1C (b)) and 2.36 nm (BL-25-25-720, Figure 1C (c)). These results further confirmed the successful incorporation of menthol into the primary mesopore channels of BMMs. Following release, BL-25-25-720-SF showed a recovery of surface area and pore volume (1302 m^2^/g and 1.65 cm^3^/g, as shown in Figure 1C (e)) together with an increase in primary pore size to 2.44 nm, indicating partial removal of menthol from the mesoporous channels.

For alcohol-thermal loading, the decrease was more pronounced: BG-640-80-60 showed a reduced surface area and pore volume of 682 m^2^/g and 0.82 cm^3^/g (Figure 1D (c)). After release, BG-640-80-60-SF exhibited a partial recovery of surface area (881 m^2^/g, as shown in Figure 1D (e)) and an increase in primary pore size (from 2.34 to 2.61 nm). However, the pore volume decreased slightly from 0.82 to 0.76 cm^3^/g and the intraparticle mesopore contribution became negligible, suggesting that the larger mesopore network may partially collapse or become blocked during the release process.

**Figure 2:**
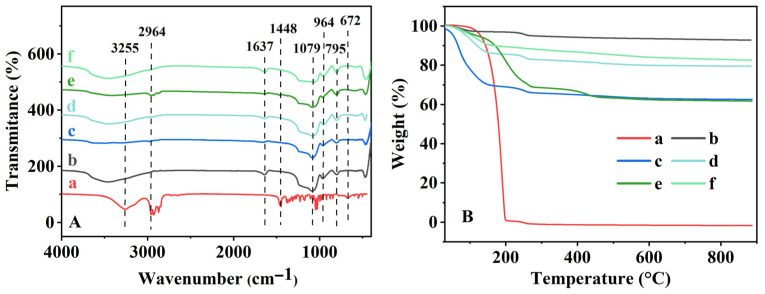
FT-IR spectra (**A**) and TG profiles (**B**) of menthol (a), BMMs (b), BL-25-25-720 (c), BL-25-25-720-SF (d), BG-640-80-60 (e), and BG-640-80-60-SF (f).

The FT-IR spectra of representative samples are presented in Figure 2A. For pure menthol (Figure 2A (a)), the bands at 3255, 2964, and 1448 cm^−1^ were assigned to -OH vibration, C-H stretching, and C-H deformation, respectively. Another that appeared at 672 cm^−1^ was ascribed to the cyclo-skeleton vibration. In contrast, BMMs (Figure 2A (b)) displayed the characteristic bands at 1637, 1079, 964, and 795 cm^−1^, which could be attributed to -OH, Si-O-Si, Si-OH, and Si-O, vibration, respectively [53]. After loading, both BL-25-25-720 (Figure 2A (c)) and BG-640-80-60 (Figure 2A (e)) showed the coexistence of menthol- and BMMs-related bands, supporting the successful incorporation of menthol. Importantly, menthol-related peaks remained detectable in the released samples (BL-25-25-720-SF in Figure 2A (d) and BG-640-80-60-SF in Figure 2A (f)), indicating residual menthol within the pores even after release, consistent with the XRD results (Figure 1A,B (e)). Scheme S1 in the ESI section illustrated the formation of the Si-O–menthyl via condensation between the -OH groups of menthol and the surface -OH groups of the BMMs. However, the characteristic peak around 1079 cm^−1^ was unobvious in the FT-IR spectra (as shown in Figure 2A), which may overlap with the strong and broad peak of Si-O-Si groups.

The TG curves of the menthol-loaded and -released BMMs prepared via the normal-temperature loading method and alcohol-thermal loading method are shown in Figure 2B. As can be seen, the pure menthol showed a dominant mass loss in the range of 30–200 °C (Figure 2B (a)), corresponding to volatilization/decomposition. For pure BMMs (Figure 2B (b)), two minor mass losses (3.1% and 2.1%) were observed, which can be attributed to the removal of physically adsorbed water (30–200 °C) and the dehydration/condensation of surface hydroxyl groups (200–300 °C). After normal-temperature loading, the mass loss associated with menthol was ~28.0% for BL-25-25-720 (Figure 2B (c)), while the corresponding released sample BL-25-25-720-SF shows a reduced mass loss of 11.3% (Figure 2B (d)), indicating partial retention of menthol after release (estimated residual fraction ~32.7%). For alcohol-thermal prepared BG-640-80-60 (Figure 2B (e)), the menthol-related weight loss extended from 30 to 200 °C (pure menthol) to approximately 30–300 °C, suggesting enhanced thermal stability arising from stronger adsorption interactions in the confined mesopores [54]. An additional weight-loss feature between 300 and 500 °C may be associated with minor byproducts generated during menthol thermal degradation. Quantitatively, the menthol-associated mass losses were ~26.3% for BG-640-80-60 (Figure 2B (e)) and ~6.9% for BG-640-80-60-SF (Figure 2B (f)), corresponding to an estimated residual menthol fraction of ~20.8% after release. As can be seen in Figure S3 of the ESI section, the DTG curves presented three peaks observed at 49, 193 and 259 °C for menthol (Figure S3A (insert)), as well as 259 °C for BMMs (Figure S3A (a)), corresponding to menthol decomposition and removal of the adsorbed physicochemical water, respectively. Comparably, two peaks appeared at 77 and 259 °C for BL-25-25-720 (Figure S3A (b)), as well as at 200, 265, and 430 °C for BG-640-80-60 (Figure S3A (d)), suggesting the successful loading of menthol into the mesoporous channels of the BMMs matrix. However, BL-25-25-720-SF (Figure S3A (c)) and BG-640-80-60-SF (Figure S3A (e)) presented almost the same DTG profiles with two peaks at 116 and 259 °C, implying partial residues of the loaded menthol in the mesoporous channels of the BMMs matrix. These phenomena were consistent with the demonstrations of the XRD patterns (Figure 1A,B) and the FT-IR spectra (Figure 2A). Meanwhile, the DTA curves of various samples were also presented in Figure S3B of the ESI section, showing the same as the DTG profiles.

**Figure 3:**
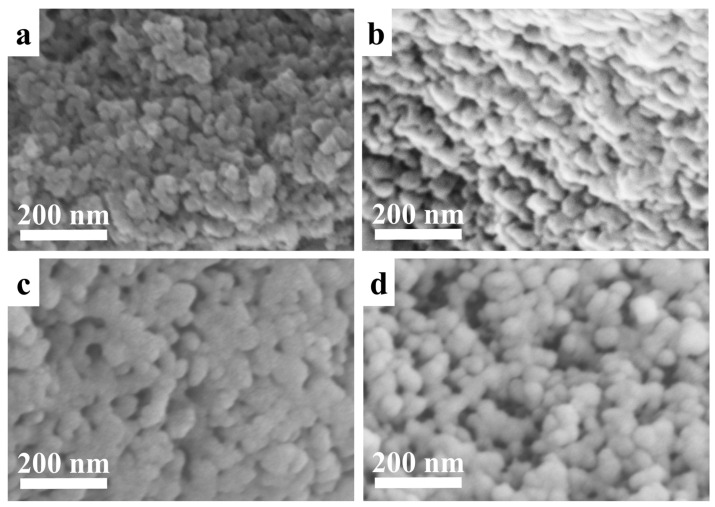
SEM images of BL-25-25-720 (**a**), BL-25-25-720-SF (**b**), BG-640-80-60 (**c**), and BG-640-80-60-SF (**d**).

**Figure 4:**
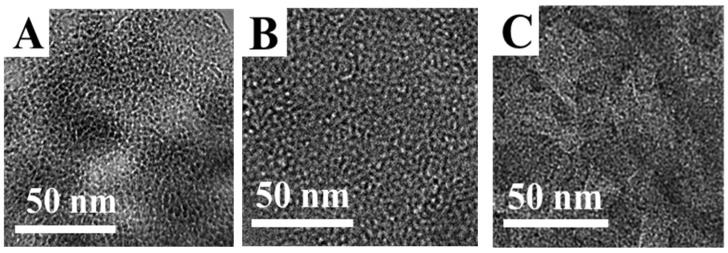
TEM images of (**A**) BMMs, (**B**) BG-640-80-60, and (**C**) BG-640-80-60-SF.

As shown in Figure S4, the BMMs consist of spherical particles with an average size of around 58 nm. After menthol loading and release, all samples (Figure 3) retained similar morphologies and particle sizes, indicating that menthol loading and the subsequent release process did not noticeably alter the particle-level structures. As shown in Figure 4, the BMMs (Figure 4A) exhibited abundant mesoporous structures with a pore size of around 2.5 nm. However, the pore size of the menthol-loaded BMMs (BG-640-80-60) (Figure 4B) decreased slightly compared to that of BMMs, indicating that menthol was loaded into the mesoporous channels of the BMMs. Meanwhile, the pore size of the menthol-released BMMs (BG-640-80-60-SF) (Figure 4C) seemly became smaller and more uneven than that of BMMs. Obviously, the mesopore features of the BMMs before menthol loading and after menthol release remained almost unchanged, being consistent with the demonstrations of the XRD patterns (Figure 1), and showing an excellent stability.

**Figure 5:**
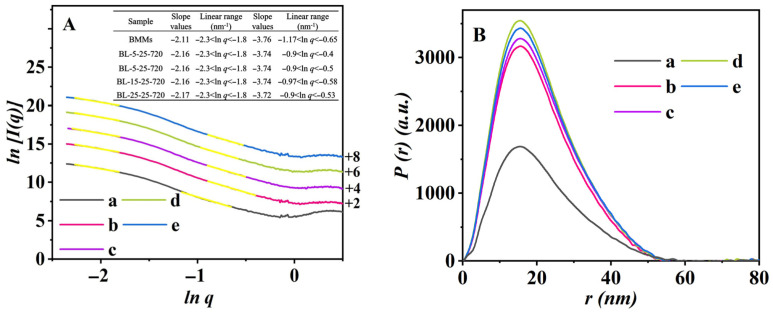
Ln-Ln plots (**A**) and PDDF profiles (**B**) of BMMs (a), BL-5-25-720 (b), BL-10-25-720 (c), BL-15-25-720 (d), and BL-25-25-720 (e). Notes: (1) Various parameters were inserted in (**A**). (2) Vertical shift values of the SAXS scattering profile (**A**) are presented in Y axis. (3) Linear fitting regions are highlighted, and the corresponding slopes were obtained by least-squares power-law fitting.

The Ln-Ln plots of scattering intensity *J*(*q*) versus *q* derived from SAXS patterns are shown in Figure 5. As can be seen, these scattering curves all showed a straight line in low *q* and high *q* regions with a high correlation coefficient (*R*^2^ > 0.996), suggesting the appearance of mass fractal and surface fractal characteristics. With increasing menthol concentrations in the normal-temperature-loaded samples, the *D_m_* values increased from 2.11 for pristine BMMs (Figure 5A (a)) to 2.16 for BL-5-25-720 (Figure 5A (b)), BL-10-25-720 (Figure 5A (c)), and BL-15-25-720 (Figure 5A (d)), and further to 2.17 for BL-25-25-720 (Figure 5A (e)). Similarly, the *D_s_* values increased from 2.24 (BMMs) to 2.26 for BL-5-25-720/BL-10-25-720/BL-15-25-720 and to 2.29 for BL-25-25-720. These trends indicated that menthol loading drives the silica network toward a more compact mass fractal organization and increases surface roughness, consistent with progressive mesopore filling.

In addition, the PDDF profiles, as shown in Figure 5B, remained largely symmetric for all samples, suggesting that spherical particle morphology was maintained before and after menthol loading [55]. The intersection of the PDDF curves with the baseline yielded a maximum particle dimension (*D_max_*) of approximately 58 nm for both BMMs and menthol-loaded BMMs, in good agreement with SEM observations (Figure 3 and Figure S4).

Figure S5 presents the ^1^H NMR spectra of the pure menthol solution and menthol-containing solution deriving from BG-640-80-60. As can be seen in Figure S5B, the menthol-containing solution deriving from BG-640-80-60 exhibited the same characteristic peaks as pure menthol (Figure S5A), indicating that the molecular structures of menthol remained unchanged during the loading process.

### 3.2. Menthol-Loading and Release Performances

**Figure 6:**
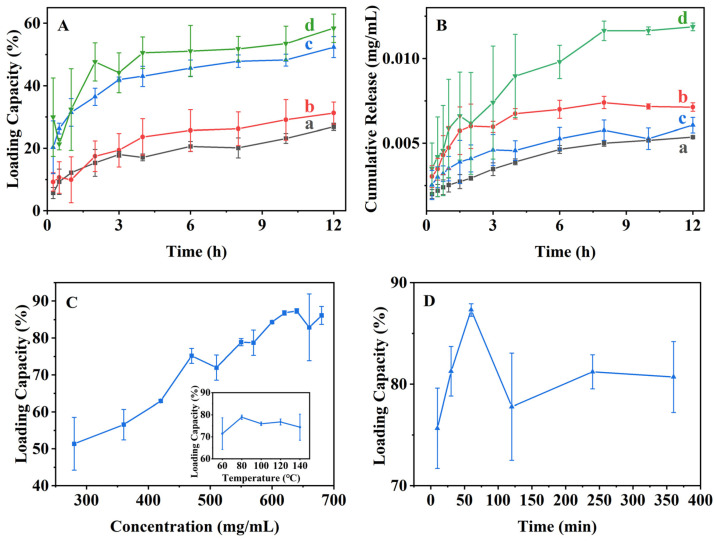
Menthol loading (**A**) and releasing (**B**) performances via normal-temperature loading method of BL-5-25-720 (a), BL-10-25-720 (b), BL-15-25-720 (c), and BL-25-25-720 (d). Menthol-loading behaviors of alcohol-thermal loading method prepared samples at different menthol concentrations and temperatures (insert) (**C**), as well as loading times (**D**).

Figure 6A presents the menthol-loading profiles of various samples prepared via the normal-temperature loading method. As can be seen, all samples exhibited similar uptake kinetics, reaching adsorption equilibrium within approximately 4 h. In contrast, the equilibrium loading capacity increases monotonically with increasing menthol concentrations, attaining a maximum value of 58% at a concentration of 25 mg·mL^−1^. These results indicated that menthol loading in the normal-temperature loading method was primarily governed by concentration-dependent diffusion into the mesoporous channels.

The corresponding menthol release profiles in ethanol are shown in Figure 6B. For all samples, an initial rapid release occurred within the first 1.5 h, followed by a slower release stage until equilibrium was reached after approximately 8 h. Notably, the burst release within the first 2 h was relatively weak, which can be attributed to the confined diffusion of menthol within the mesoporous channels. The initial release stage was mainly associated with physically adsorbed menthol located on or near the external surfaces of BMMs, whereas the subsequent slow release arose from menthol molecules confined within the mesopores and interacting with surface hydroxyl groups. This behavior was consistent with the diffusion-limited transport revealed later by molecular dynamics simulations. In addition, the cumulative release amount increased with increasing menthol loading, reflecting a concentration-driven release process.

The menthol-loading performances of samples prepared via the alcohol-thermal loading method were further investigated. As can be seen in Figure 6C, the loading capacity increased markedly with increasing menthol concentrations and reached a maximum value of 87% at 640 mg·mL^−1^, significantly surpassing that achieved via the normal-temperature loading route. Figure 6C (insert) shows the effect of temperature, where the loading capacity initially increased and then slightly decreased with increasing temperature. Similarly, Figure 6D demonstrates that the loading capacity increased with time during the early stage and reached a maximum at 60 min, followed by a gradual decline at longer times.

These behaviors can be explained by diffusion-controlled adsorption within the mesoporous channels. During the initial stage (10–60 min), the enhanced molecular mobility promotes the efficient diffusion and adsorption of menthol within the pores. At longer times, the excessive menthol accumulation near pore entrances likely blocks internal diffusion pathways, limiting further adsorption and resulting in a decrease in overall loading capacity. Based on these results, optimal loading conditions were identified as 80 °C, 640 mg·mL^−1^, and 60 min, under which a maximum loading capacity of 87% was achieved.

**Figure 7:**
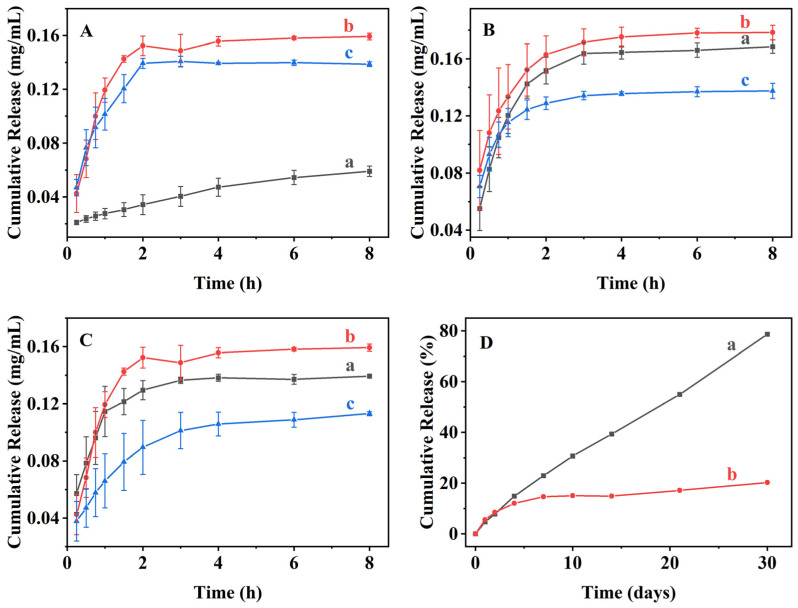
Menthol-releasing behaviors of the related samples prepared via alcohol-thermal loading method at different menthol concentrations (**A**): BG-280-80-60 (a), BG-640-80-60 (b), BG-680-80-60 (c); loaded temperature (**B**): BG-550-60-60 (a), BG-550-80-60 (b), BG-550-100-60 (c); loaded times (**C**): BG-640-80-30 (a), BG-640-80-60 (b), BG-640-80-120 (c). Meanwhile, Panel (**D**) shows the atmospheric release profiles of pure menthol (a) and BG-640-80-60 (b).

Figure 7 presents the menthol release behaviors of alcohol-thermal-loaded samples. As shown in Figure 7A, all samples exhibited a two-stage release profile consisting of an initial rapid release within 0–3 h, followed by a slower release stage until equilibrium was reached. The initial stage was attributed to the release of physically adsorbed menthol on the external surfaces of BMMs, whereas the latter stage corresponded to the diffusion-limited release of menthol confined within the mesoporous channels.

For samples loaded at lower concentrations, such as BG-280-80-60 (Figure 7A (a)), equilibrium was not reached within 8 h, which was attributed to strong hydrogen-bonding interactions between menthol molecules and surface hydroxyl groups inside the mesopores, hindering molecular diffusion. In contrast, samples loaded at higher concentrations (BG-640-80-60 and BG-680-80-60, as shown in Figure 7A (b) and (c)) reached equilibrium within 4 h due to the formation of steeper concentration gradients that facilitated faster diffusion. However, the equilibrium release percentage decreased from 5.44% (BG-280-80-60, as shown in Figure 7A (a)) to 2.31% (BG-640-80-60, as shown in Figure 7A (b)) and 2.18% (BG-680-80-60, as shown in Figure 7A (c)), reflecting the higher loading densities achieved under these conditions.

Similarly, increasing the loading temperature led to a decrease in equilibrium release amount (Figure 7B), while variation in loading time produced comparable release profiles with a minimum equilibrium release observed at 60 min (Figure 7C). These trends were consistent with the corresponding loading behaviors shown in Figure 6C (insert),D.

Under atmospheric conditions (Figure 7D), pure menthol exhibited rapid volatilization, with a cumulative release of 78.7% after 30 days (Figure 7D (a)). In contrast, BG-640-80-60 showed a significantly suppressed release, characterized by an initial fast release stage (0–4 days) followed by a slow release phase, reaching only 20.3% after 30 days (Figure 7D (b)). This pronounced retardation demonstrated the effectiveness of BMMs in stabilizing volatile menthol and highlighted their potential as controlled release carriers.

### 3.3. Adsorption Kinetics

The adsorption kinetics of menthol were analyzed using PFO, PSO, and IPD models, respectively, as shown in Figure S6 of the ESI section, with fitting parameters summarized in Table S2. These models are conducive to predicting the equilibrium rate of the menthol adsorption and clarifying the adsorption mechanism. For normal-temperature-loaded BL-25-25-720, the PSO model provided the best fit, as evidenced by the highest correlation coefficient (*R*^2^), indicating that adsorption kinetics were dominated by surface interactions combined with diffusion within the hierarchical mesoporous network. For alcohol-thermal-loaded BG-640-80, both the PFO and PSO models yielded relatively high and comparable *R*^2^ values (0.89 and 0.88), suggesting that menthol adsorption involved a combination of physisorption and weak chemisorption. In contrast, the IPD model exhibited a significantly lower *R*^2^ value (0.42), indicating that intraparticle diffusion was not the sole rate-limiting step for the overall adsorption process [56]. These results confirmed that the bimodal mesopore architecture enabled efficient mass transport and high adsorption capacity.

### 3.4. Adsorption Isotherms

The adsorption isotherms were further demonstrated using Langmuir, Freundlich, Temkin, and D–R models (Figure S7), with fitting parameters summarized in Table 1. For normal-temperature-loaded samples (Figure S7A and Table 1), all four models exhibited good fitting quality (*R*^2^ > 0.9), with the Langmuir model showing the highest correlation coefficient. This indicated that menthol adsorption predominantly occurred via monolayer coverage within the mesoporous channels. The Temkin model further suggested that intermolecular interactions played a significant role in the adsorption process.

For alcohol-thermal-loaded samples, multiple isotherm models provided good fits, reflecting a cooperative adsorption mechanism (Figure S7B and Table 1). The high *R*^2^ value obtained from the D–R model suggested that pore-filling was the dominant adsorption mechanism, rather than simple surface monolayer adsorption. Meanwhile, the Langmuir and Freundlich models indicated the coexistence of uniform adsorption sites and surface heterogeneity within the mesoporous framework. A negative *n* value in Freundlich models indicates the interactions among the adsorbed molecules [57]. Specifically, menthol molecules were initially adsorbed onto the silanol sites on the mesoporous surfaces of BMMs via van der Waals forces, which was conducive to facilitating the formation of multilayer adsorption. These results collectively demonstrated that alcohol-thermal loading promoted more efficient mesopore utilization and deeper penetration of menthol into the hierarchical mesoporous structure. These differences account for the high loading capacity achieved by alcohol-thermal loading, indicating that alcohol-thermal loading may be a suitable strategy for stabilizing and controlling menthol delivery within mesoporous systems.

### 3.5. Thermodynamics of Menthol Adsorption

The thermodynamic parameters governing menthol adsorption on BMMs, including the Δ*G*, Δ*H*, and Δ*S* values, are summarized in Table 2. For both normal-temperature and alcohol-thermal loading, the positive Δ*G* values indicated that menthol adsorption under the investigated conditions was thermodynamically non-spontaneous [58]. For normal-temperature loading, the utilization of the high-concentration menthol solutions (5–30 mg/mL) is conducive to generating a large concentration gradient that drives molecular diffusion into the mesopore channels of BMMs, effectively overcoming thermodynamic limitations. In alcohol-thermal loading, the elevated temperature and pressure is conducive to facilitating more rapid diffusion of menthol molecules into the mesopore channels, thereby overcoming thermodynamic barriers. Nevertheless, the temperature dependence of Δ*G* values revealed distinct adsorption behaviors for two loading routes. Increasing temperature favored menthol adsorption in the normal-temperature loading system, whereas it had an adverse effect on adsorption in the alcohol-thermal loading system.

For normal-temperature loading adsorption, the positive Δ*H* value (9.48 kJ·mol^−1^) indicated an endothermic process, which was likely associated with the disruption of the weak intermolecular interactions between menthol molecules and solvent prior to adsorption. The positive Δ*S* value (8.30 J·mol^−1^·K^−1^) suggested an increase in system randomness during adsorption, possibly due to the redistribution of menthol molecules within the mesoporous channels and changes in interfacial ordering at the solid–liquid interface. These thermodynamic features were characteristic of a physically driven adsorption process dominated by weak interactions, being consistent with multilayer adsorption behavior within the mesoporous structures.

In contrast, menthol adsorption via the alcohol-thermal loading route exhibited an exothermic nature, as evidenced by the negative Δ*H* value (−5.89 kJ·mol^−1^). This behavior can be attributed to the formation of weak van der Waals interactions between menthol molecules and surface hydroxyl groups on BMMs, resulting in the lower volatility of the alcohol-thermal-loaded sample than that of pure menthol during atmospheric release. The relatively small absolute value of Δ*H* further confirmed that the adsorption process was governed by physisorption rather than chemisorption. Notably, the negative Δ*S* value (−57.88 J·mol^−1^·K^−1^) indicated the increased molecular ordering at the solid interfaces, reflecting a more constrained arrangement of the menthol molecules within the mesopores during alcohol-thermal loading adsorption. Consequently, despite the exothermic nature of the process, the unfavorable Δ*S* value constitutes a major thermodynamic barrier to spontaneous adsorption [59]. The thermodynamic data suggest that alcohol-thermal loading enables a more stable and dense arrangement of the loaded menthol, providing a theoretical basis for developing high-dose formulations.

### 3.6. Release Kinetics of Menthol

The release kinetics of menthol from BMMs prepared via the normal-temperature method and alcohol-thermal loading method were demonstrated using first-order, Higuchi, and K–P models, as shown in Figures S8 and S9. The corresponding kinetic parameters are summarized in Table 3 and Table S3. For all normal-temperature-loaded samples, the K–P model yielded the highest *R*^2^ value (> 0.96), indicating that menthol release predominantly followed a non-Fickian diffusion mechanism. This behavior was mainly governed by the diffusion of menthol molecules through the interconnected mesoporous channels of BMMs, consistent with previous reports on mesoporous carriers [60]. In contrast, the relatively low *R*^2^ values obtained from the first-order and Higuchi models suggested that these models were inadequate to describe the release behavior of the normal-temperature-loaded samples.

For alcohol-thermal-loaded samples, the release mechanism exhibited a clear dependence on the initial loading concentration. At lower menthol loading levels (e.g., BG-280-80-60), the release profiles were best described by the K–P model, indicating diffusion-controlled release from the mesoporous framework. However, samples prepared at higher loading concentrations (BG-640-80-60 and BG-680-80-60) followed first-order kinetics, implying that the release process was dominated by a concentration gradient-driven desorption mechanism. As discussed earlier, high menthol loading led to near-saturation conditions within the mesopores, resulting in multilayer adsorption and partial pore blockage, which restricted diffusion and rendered desorption the rate-limiting step.

Similarly, alcohol-thermal-loaded samples prepared under different temperatures and loading durations also exhibited first-order release behavior, further confirming that menthol release under saturated adsorption conditions was primarily controlled by desorption rather than simple diffusion. These findings demonstrated a transition in the release mechanism from diffusion-dominated to desorption-limited behavior as the menthol loading level increased.

### 3.7. MD Simulation

The MD simulations revealed clear differences in menthol diffusion and interfacial interactions within mesopores of BMMs for alcohol-thermal and normal-temperature loading. Based on the MSD profile (as shown in Figure S10), the diffusion coefficient of menthol in the alcohol-thermal loading system (1.01 × 10^−6^ m^2^·s^−1^) was approximately five times higher than that in the normal-temperature loading system (2.12 × 10^−7^ m^2^·s^−1^), reflecting faster diffusion and more uniform distribution of the menthol loaded via the alcohol-thermal loading method. In contrast, the slower diffusion in the normal-temperature loading method tended to cause localized accumulation or partial pore blockage, therefore reducing the loading capacity, consistent with the experimental loading results (Figure 6A).

**Figure 8:**
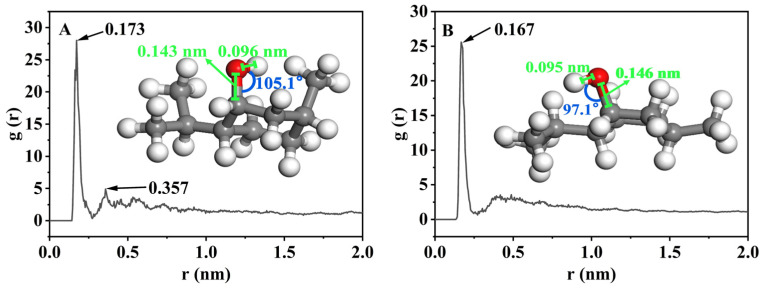
RDF profiles of normal-temperature (**A**) and alcohol-thermal (**B**) loading for menthol in the mesoporous channel models of BMMs and corresponding dominant configuration (inset).

RDF profiles between oxygen atoms of menthol and hydrogen atoms of surface Si-OH in BMMs showed two characteristic peaks in the normal-temperature loading system (Figure 8A), located at approximately 0.173 nm and 0.357 nm, respectively. In contrast, only one characteristic peak in the alcohol-thermal loading system (Figure 8B) was observed at about 0.167 nm. As reported by Mirhosseini et al. [61], the peak that appeared at around 0.17 nm was attributed to the hydrogen bonds formed between menthol and surface Si-OH in BMMs. Furthermore, its intensity in the normal-temperature loading system was slightly higher than that in the alcohol-thermal loading system, indicating the presence of stronger hydrogen bonds in the normal-temperature loading system than in the alcohol-thermal loading system.

Based on the RDF data deriving from normal-temperature and alcohol-thermal loading systems, the adsorption configuration diagrams of menthol molecules at various adsorption sites were also simulated. The free-state configuration of menthol is shown in Figure S11A, with a bond angle of 107.2°, a C-O bond length of 0.144 nm, and an O-H bond length of 0.097 nm. Meanwhile, the peak intensity at 0.173 nm in the normal-temperature loading system (Figure 8A) was significantly stronger than the peak at 0.357 nm, indicating the presence of the primary adsorption configuration of menthol molecules loaded on the mesoporous surfaces of BMMs, as shown in Figure 8A (inset). This configuration exhibited a bond angle of 105.1°, a C-O bond length of 0.143 nm, and an O-H bond length of 0.096 nm, all of which were slightly decreased compared to the free state of menthol (Figure S11A). Meanwhile, the simulated configuration at 0.357 nm in the normal-temperature loading system is shown in Figure S11B. Comparably, only one characteristic peak in the alcohol-thermal loading system was observed at 0.167 nm, suggesting the appearance of a single adsorption configuration of menthol molecules, as shown in Figure 8B (inset). This configuration (Figure 8B (inset)) presented a bond angle of 97.1°, a C-O bond length of 0.146 nm, and an O-H bond length of 0.095 nm, showing a significant decrease in the bond angle and a slight decrease in C-O bond length, compared to the free state of menthol (Figure S11A).

## 4. Conclusions

This work presents a systematic and mechanistically grounded comparison of the normal-temperature and alcohol-thermal loading strategies for the encapsulation of volatile menthol using BMMs as a carrier. Through rational optimization of alcohol-thermal loading parameters, an exceptionally high menthol loading capacity of 87% was achieved, substantially exceeding the maximum value of 58% obtained via conventional normal-temperature loading. Comprehensive structural and physicochemical characterizations confirmed that the hierarchical mesoporous architecture of BMMs remained intact throughout loading and release, while SAXS patterns revealed the increased structural compactness and surface roughness upon menthol incorporation. Release kinetics uncovered a critical mechanism transition in the alcohol-thermal-loaded samples, from diffusion-controlled release at low loadings to concentration gradient-driven desorption at high loadings. This transition, evidenced by the shift from K–P to first-order kinetics, arose from adsorption saturation and partial pore blockage, which became the dominant barriers to release. MD simulations provided direct molecular-level insight into this behavior, showing that menthol diffusion in the alcohol-thermal loading system was approximately five times faster than in the normal-temperature loading system. Meanwhile, the normal-temperature loading system exhibited stronger hydrogen bonds between the menthol and surface Si-OH of BMMs than in the alcohol-thermal loading system. The practical relevance of these mechanistic findings was validated by long-term atmospheric release tests, where alcohol-thermal-loaded BMMs (BG-640-80-60) exhibited a markedly sustained release profile, with only 20.3% menthol released over 30 days compared to 78.7% for pure menthol. Collectively, these results demonstrated that alcohol-thermal loading enabled superior encapsulation efficiency and controlled release by synergistically enhancing molecular diffusion, weakening interfacial constraints, and ensuring uniform pore-filling. These demonstrations established alcohol-thermal loading as a powerful and generalizable strategy for the stabilization and controlled delivery of volatile compounds in mesoporous systems, offering broad potential for applications in pharmaceuticals, flavor preservation, and functional materials.

## Figures and Tables

**Table 1:** Summaries of the isotherm parameters for menthol adsorption on BMMs obtained by fitting the experimental data to Langmuir, Freundlich, Temkin, and D–R models for normal-temperature and alcohol-thermal loading.

Models	Parameters	Normal-Temperature Loading	Alcohol-Thermal Loading
Langmuir	*q_m_*	3976.1	9103.8
	*K_L_*	2.5558 × 10^−5^	1.2267 × 10^−21^
	*R* ^2^	0.9332	0.9312
Freundlich	*K_F_*	0.7765	4.2227 × 10^−7^
	*n*	0.7521	−1.7396
	*R* ^2^	0.9186	0.9170
Temkin	*b*	672.8	6439.2
	*A*	3.6833 × 10^−4^	3.6360 × 10^−6^
	*R* ^2^	0.9547	0.9249
D–R	*K_DR_*	7.5492 × 10^7^	2.8667 × 10^11^
	*q_DR_*	1678.2	11,156.2
	*R* ^2^	0.9282	0.9501

**Table 2:** Summaries of the thermodynamic parameters for menthol adsorption on BMMs via normal-temperature and alcohol-thermal loading methods.

Loading Method	Δ*G* (kJ·mol^−1^)			Δ*H* (kJ·mol^−1^)	Δ*S* (J·mol^−1^·K^−1^)	*R* ^2^
Normal-temperature	25 °C	35 °C	45 °C	9.48	8.30	0.9758
	7.06	6.82	6.60			
Alcohol-thermal	80 °C	100 °C	120 °C	−5.89	−57.88	0.9817
	14.58	15.71	16.94			

**Table 3:** Summaries of the kinetic parameters describing menthol release from normal-temperature- and alcohol-thermal-loaded BMMs obtained by fitting the first-order, Higuchi, and K–P models.

Models	Parameters	BL-5-25-720	BL-25-25-720	BG-640-80-10	BG-640-80-60
First-order	*K*_1_	0.5100	0.3151	1.191	1.0311
	*a*	0.0051	0.0120	0.1371	0.1629
	*R* ^2^	0.8609	0.9560	0.9985	0.9850
Higuchi	*K_H_*	0.0015	0.0037	0.0488	0.061
	*c*	0.0008	0.0002	0.0308	0.027
	*R* ^2^	0.9523	0.9708	0.8141	0.7857
K–P	*K_KP_*	0.0024	0.0042	0.0933	0.1064
	*n*	0.3431	0.4523	0.2592	0.29
	*c*	8 × 10^−5^	−2 × 10^−4^	−0.0043	−0.0087
	*R* ^2^	0.9764	0.9727	0.9234	0.8629

## Data Availability

The data presented in this article is available from the Supplementary Information or an online repository.

## Supplementary Materials

The following supporting information can be downloaded at: https://www.mdpi.com/article/10.3390/nano16090545/s1, Scheme S1. Illustration of the preparation process for loading and releasing menthol. Figure S1. The MD simulation models of the front and side views of BMMs (A), and the menthol-loaded BMMs prepared by normal-temperature loading method (B) or alcohol-thermal loading method (C). Note: Red, white, yellow, and gray represent oxygen, hydrogen, silicon, and carbon, respectively. Figure S2. Pore size distribution curves of samples prepared via normal-temperature loading method (A) and alcohol-thermal loading method (B). A: BMMs (a), BL-5-25-720 (b), BL-25-25-720 (c), BL-5-25-720-SF (d), and BL-25-25-720-SF (e). B: BMMs (a), BG-640-80-10 (b), BG-640-80-60 (c), BG-640-80-10-SF (d), and BG-640-80-60-SF (e). Figure S3. DTG (A) and DTA (B) curves of BMMs (a), BL-25-25-720 (b), BL-25-25-720-SF (c), BG-640-80-60 (d), BG-640-80-60-SF (e) and menthol (inset). Figure S4. SEM image of BMMs. Figure S5. ^1^H-NMR spectra of pure menthol solution (A) and menthol-containing solution deriving from BG-640-80-60 (B) in deuterated chloroform. Figure S6. Menthol adsorption kinetics of BL-25-25-720 (A) and BG-640-80 (B) fitted using PFO, PSO, and IPD models. Figure S7. Adsorption isotherms of menthol for the normal-temperature-loaded (A) and alcohol-thermal-loaded (B) samples fitted using Langmuir, Freundlich, Temkin, and D–R models, respectively. Figure S8. Menthol-releasing kinetics of BL-5-25-720 (A), BL-10-25-720 (B), BL-15-25-720 (C), and BL-25-25-720 (D), which are fitted using first-order, Higuchi, and K–P, respectively. Figure S9. Menthol-releasing kinetics of BG-280-80-60 (A), BG-640-80-60 (B), BG-680-80-60 (C), BG-550-60-60 (D), BG-550-80-60 (E), BG-550-100-60 (F), BG-640-80-10 (G), BG-640-80-30 (H), and BG-640-80-120 (I), which are fitted using first-order, Higuchi, and K–P, respectively. Figure S10. MSD profiles for menthol molecules in the mesoporous channel models of BMMs via normal-temperature loading method (a) and alcohol-thermal loading method (b). Figure S11. Simulated configuration diagrams of free-state menthol (A) and adsorption sites at 0.357 nm in the normal-temperature loading system (B). Table S1. Summaries of the synthesis parameters of the menthol-loaded BMMs prepared via normal-temperature and alcohol-thermal loading methods. Table S2. Summaries of the kinetic parameters for menthol adsorption on BL-25-25-720 (normal-temperature loading) and BG-640-80 (alcohol-thermal loading) fitted using PFO, PSO, and IPD models, respectively. Table S3. Summaries of the kinetic parameters for menthol release from normal-temperature- and alcohol-thermal-loaded BMMs obtained by fitting the first-order, Higuchi, and K–P models, respectively.
