# Supplementary material for: Encapsulation of Menthol in Bimodal Mesoporous Silica via Normal-Temperature and Alcohol-Thermal Loading Methods for Achieving Sustained Releasing Performances
Source: Nanomaterials (Basel). 2026 Apr 29;16(9):545. doi: 10.3390/nano16090545 (PMC13165228; doi:10.3390/nano16090545)
Supplement: Supplementary file 1 [file nanomaterials-16-00545-s001.zip › nanomaterials-4266244-supplementary.pdf]

Electronic Supporting Information

Captions for Figures and Tables

Scheme S1. Illustration of the preparation process for loading and releasing menthol.

Figure S1. The MD simulation models of the front and side views of BMMs (A), and the menthol-loaded BMMs prepared by normal-temperature loading method (B) or alcohol-thermal loading method (C). Noted: Red, white, yellow, and grey represent oxygen, hydrogen, silicon, and carbon, respectively.

Figure S2. Pore size distribution curves of samples prepared via normal-temperature loading method (A) and alcohol-thermal loading method (B). A: BMMs (a), BL-5-25-720 (b), BL-25-25-720 (c), BL-5-25-720-SF (d), and BL-25-25-720-SF (e). B: BMMs (a), BG-640-80-10 (b), BG-640-80-60 (c), BG-640-80-10-SF (d), and BG-640-80-60-SF (e).

Figure S3. DTG (A) and DTA (B) curves of BMMs (a), BL-25-25-720 (b), BL-25-25-720-SF (c), BG-640-80-60 (d), BG-640-80-60-SF (e) and menthol (insert).

Figure S4. SEM image of BMMs.

Figure S5. ^{1}H-NMR spectra of pure menthol solution (A) and menthol-contained solution deriving from BG-640-80-60 (B) in deuterated chloroform.

Figure S6. Menthol adsorption kinetics of BL-25-25-720 (A) and BG-640-80 (B) fitted using PFO, PSO, and IPD models.

Figure S7. Adsorption isotherms of menthol for normal-temperature-loaded **(A)** and

alcohol-thermal-loaded **(B)** samples fitted using Langmuir, Freundlich, Temkin, and D–R models, respectively.

Figure S8. Menthol-releasing kinetics of BL-5-25-720 (A), BL-10-25-720 (B), BL-15-25-720 (C), and BL-25-25-720 (D), which are fitted using first-order, Higuchi, and K–P, respectively.

Figure S9. Menthol-releasing kinetics of BG-280-80-60 (A), BG-640-80-60 (B), BG-680-80-60 (C), BG-550-60-60 (D), BG-550-80-60 (E), BG-550-100-60 (F), BG-640-80-10 (G), BG-640-80-30 (H), and BG-640-80-120 (I), which are fitted using first-order, Higuchi, and K–P, respectively.

Figure S10. MSD profiles for menthol molecules in the mesoporous channel models of BMMs via normal-temperature loading method (a) and alcohol-thermal loading method (b).

Figure S11. Simulated configuration diagrams of free-state menthol (A) and adsorption sites at 0.357 nm in the normal-temperature loading system (B).

Table S1. Summaries of the synthesis parameters of the menthol-loaded BMMs prepared via normal-temperature and alcohol-thermal loading methods.

Table S2. Summaries of the kinetic parameters for menthol adsorption on BL-25-25-720 (normal-temperature loading) and BG-640-80 (alcohol-thermal loading) fitted using PFO, PSO, and IPD models, respectively.

Table S3. Summaries of the kinetic parameters for menthol release from normal-

temperature- and alcohol-thermal-loaded BMMs obtained by fitting the first-order, Higuchi, and K–P models, respectively.

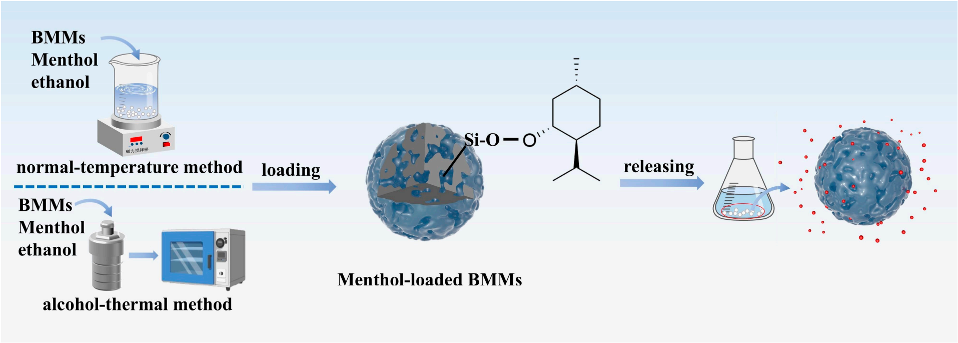

Scheme S1. Illustration of the preparation process for loading and releasing menthol.

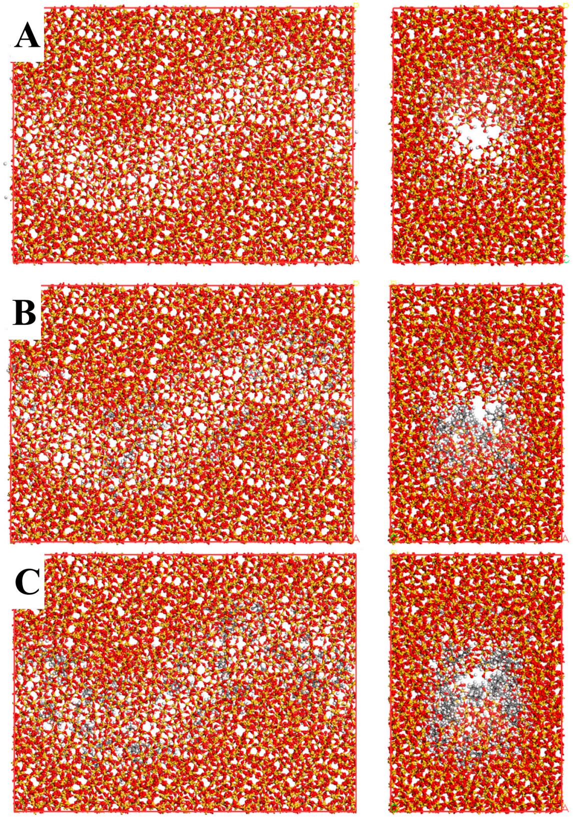

Figure S1. The MD simulation models of the front and side views of BMMs (A), and the menthol-loaded BMMs prepared by normal-temperature loading method (B) or alcohol-thermal loading method (C). Noted: Red, white, yellow, and grey represent oxygen, hydrogen, silicon, and carbon, respectively.

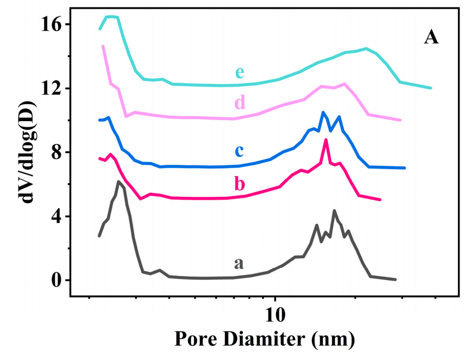

Figure S2. Pore size distribution curves of samples prepared via normal-temperature loading method (A) and alcohol-thermal loading method (B). A: BMMs (a), BL-5-25-720 (b), BL-25-25-720 (c), BL-5-25-720-SF (d), and BL-25-25-720-SF (e). B: BMMs (a), BG-640-80-10 (b), BG-640-80-60 (c), BG-640-80-10-SF (d), and BG-640-80-60-SF (e).

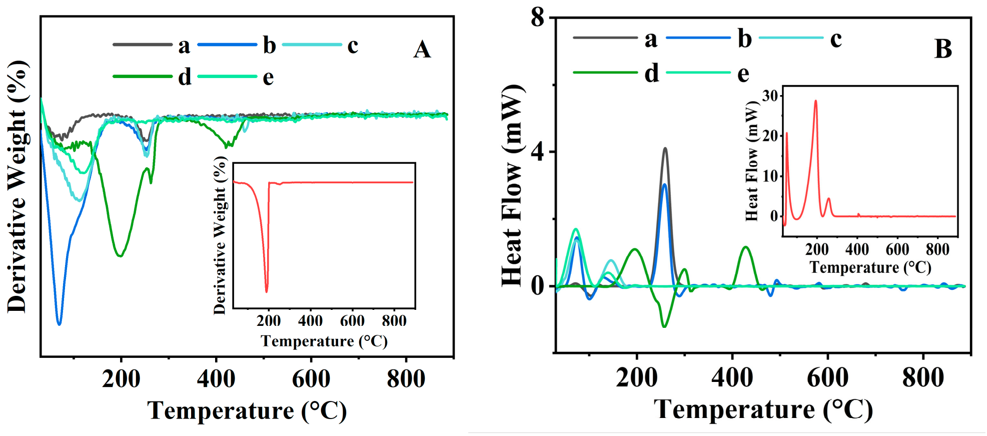

Figure S3. DTG (A) and DTA (B) curves of BMMs (a), BL-25-25-720 (b), BL-25-25-720-SF (c), BG-640-80-60 (d), BG-640-80-60-SF (e) and menthol (insert).

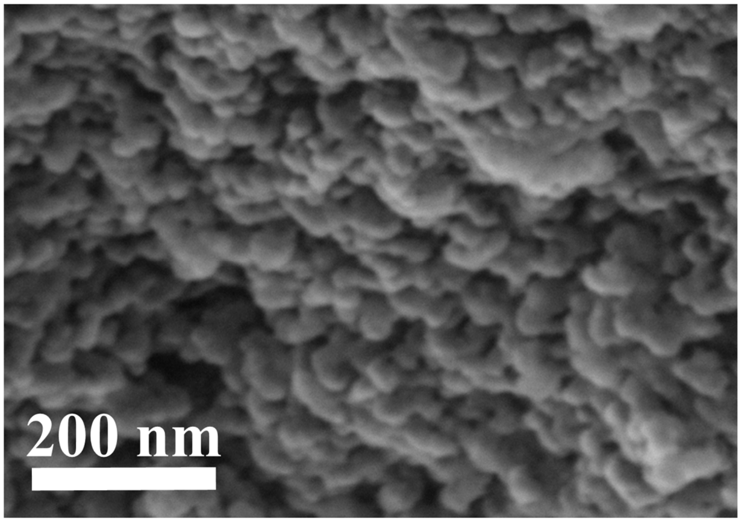

Figure S4. SEM image of BMMs.

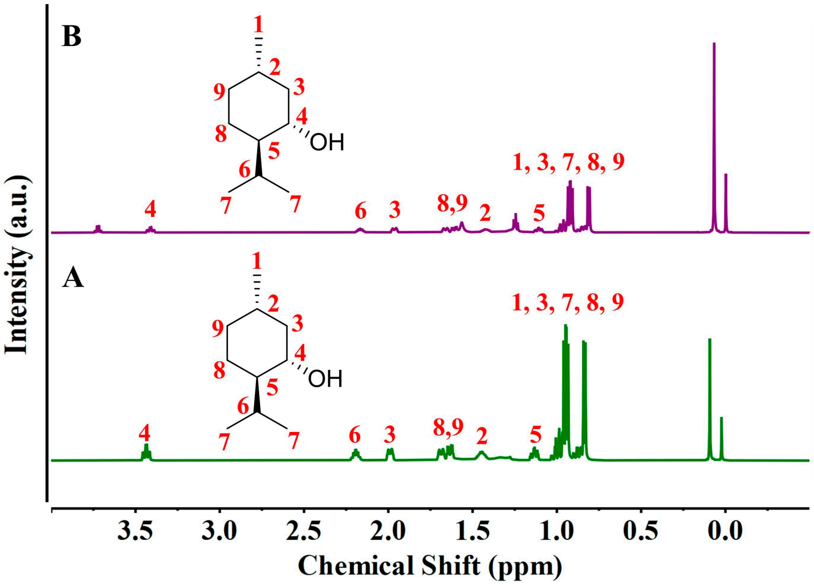

Figure S5. ^{1}H-NMR spectra of pure menthol solution (A) and menthol-contained solution deriving from BG-640-80-60 (B) in deuterated chloroform.

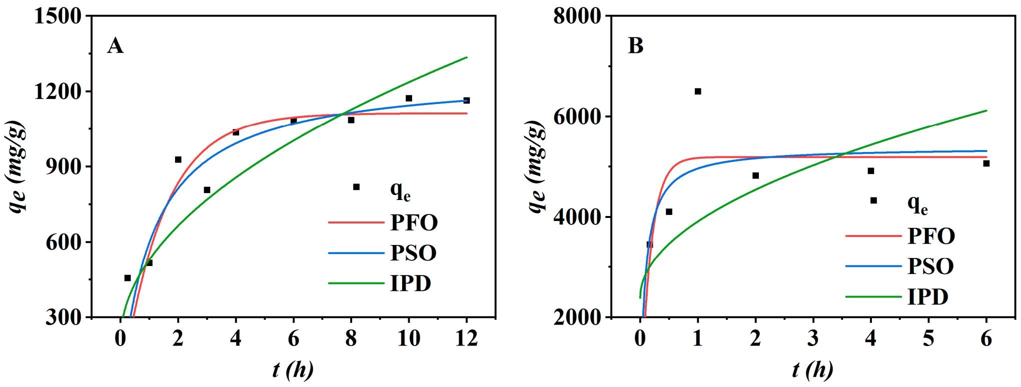

Figure S6. Menthol adsorption kinetics of BL-25-25-720 (A) and BG-640-80 (B) fitted using PFO, PSO, and IPD models.

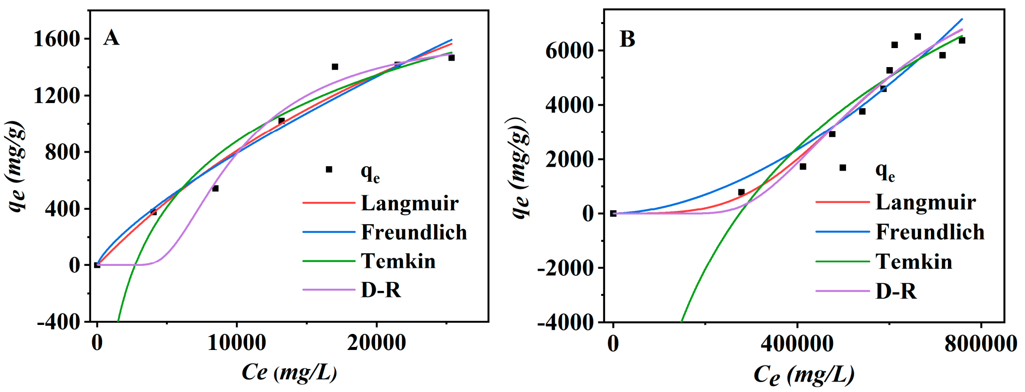

Figure S7. Adsorption isotherms of menthol for normal-temperature-loaded **(A)** and alcohol-thermal-loaded **(B)** samples fitted using Langmuir, Freundlich, Temkin, and D–R models, respectively.

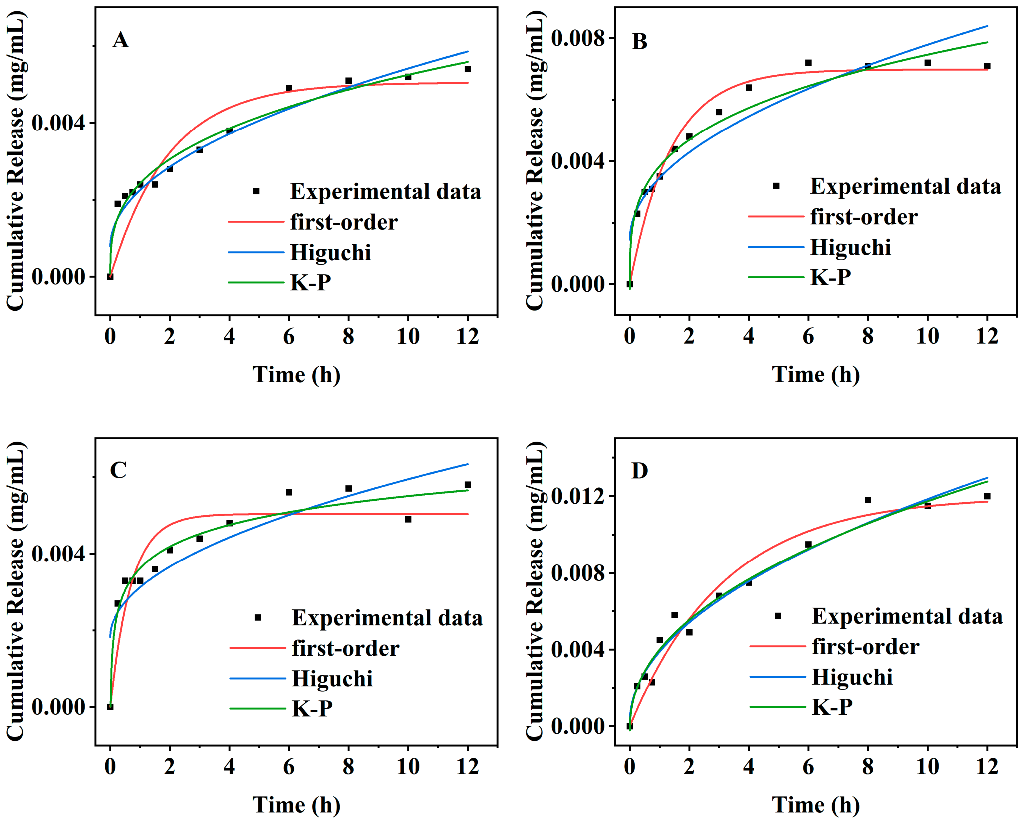

Figure S8. Menthol-releasing kinetics of BL-5-25-720 (A), BL-10-25-720 (B), BL-15-25-720 (C), and BL-25-25-720 (D), which are fitted using first-order, Higuchi, and K–P, respectively.

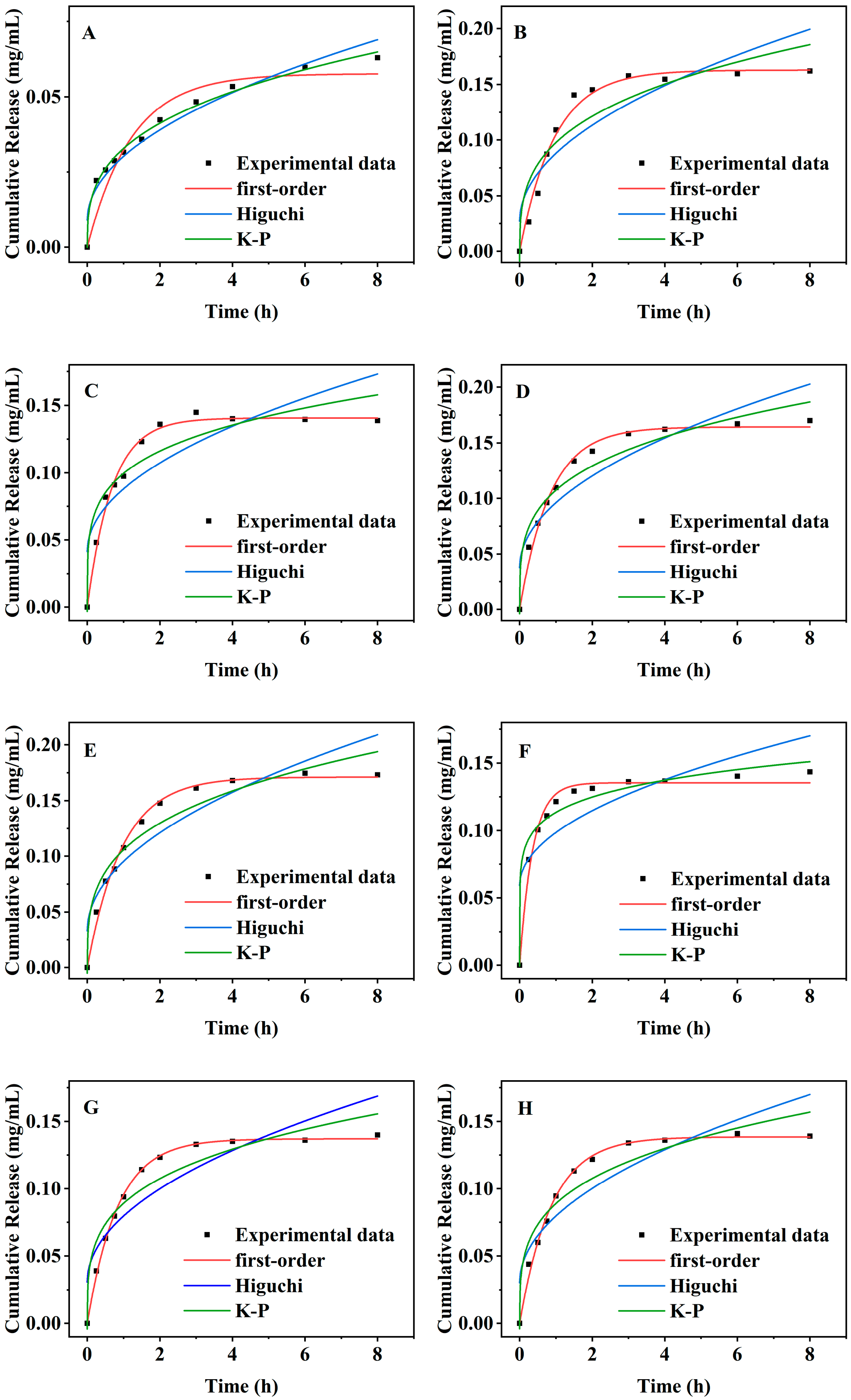
A
B
C
D
E
F
G
H
Cumulative Release (mg/mL)
Time (h)
Experimental data
first-order
Higuchi
K-P
0.00
0.05
0.10
0.15
0.20
0
2
4
6
8

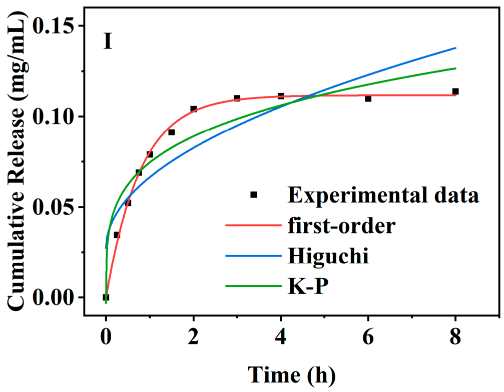

Figure S9. Menthol-releasing kinetics of BG-280-80-60 (A), BG-640-80-60 (B), BG-680-80-60 (C), BG-550-60-60 (D), BG-550-80-60 (E), BG-550-100-60 (F), BG-640-80-10 (G), BG-640-80-30 (H), and BG-640-80-120 (I), which are fitted using first-order, Higuchi, and K–P, respectively.

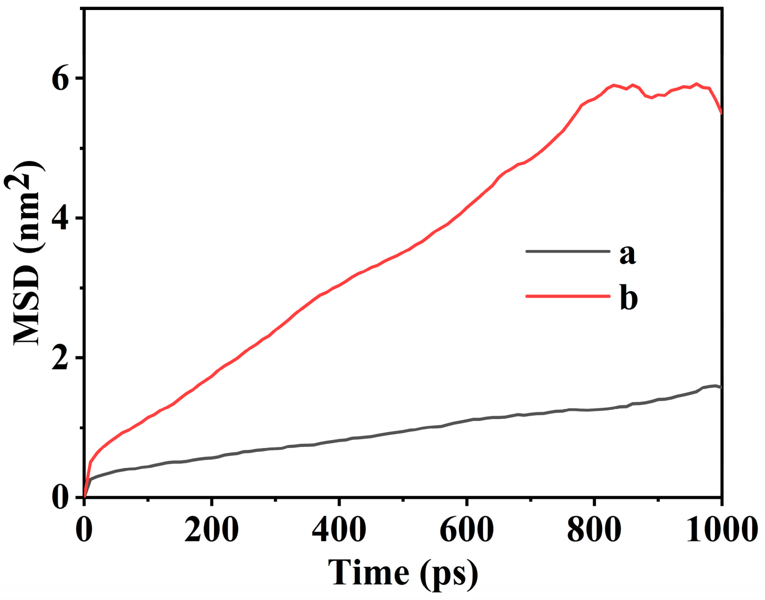

Figure S10. MSD profile for menthol molecules in the mesoporous channel model of BMMs via normal-temperature loading method (a) and alcohol-thermal loading method (b).

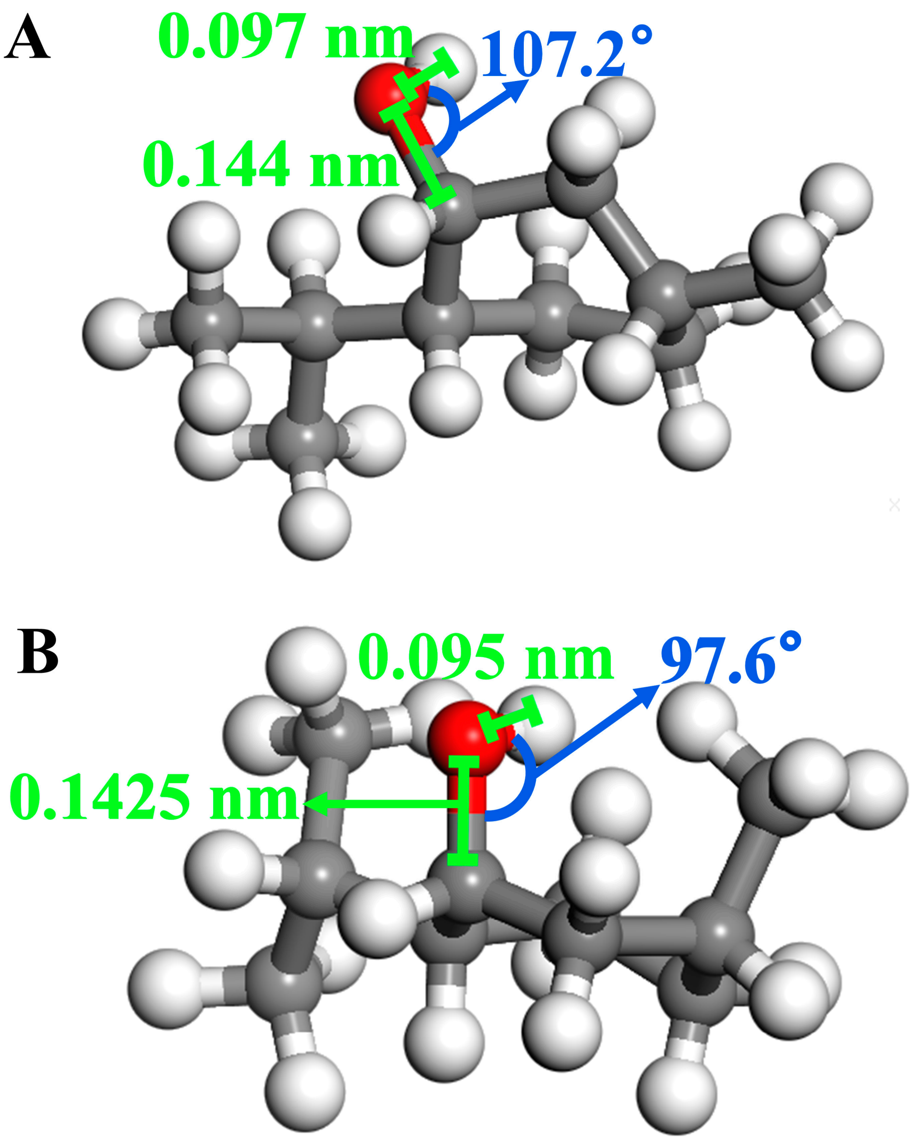

Figure S11. Simulated configuration diagrams of free-state menthol (A) and adsorption sites at 0.357 nm in the normal-temperature loading system (B).

Table S1. Summaries of the synthesis parameters of the menthol-loaded BMMs prepared via normal-temperature and alcohol-thermal loading methods.

**Methods**	**Samples**	**Menthol Concentration ($mg·mL^{-1}$)**	**Temperature (°C)**	**Time (min)**
Normal-temperature loading method	BL-5-25-720	5	25	720
	BL-10-25-720	10	25	720
	BL-15-25-720	15	25	720
	BL-20-25-720	20	25	720
	BL-25-25-720	25	25	720
	BL-30-25-720	30	25	720
Alcohol-thermal loading method	BG-280-80-60	280	80	60
	BG-360-80-60	360	80	60
	BG-420-80-60	420	80	60
	BG-470-80-60	470	80	60
	BG-510-80-60	510	80	60
	BG-550-80-60	550	80	60
	BG-570-80-60	570	80	60
	BG-600-80-60	600	80	60
	BG-620-80-60	620	80	60
	BG-640-80-60	640	80	60
	BG-660-80-60	660	80	60
	BG-680-80-60	680	80	60
	BG-550-60-60	550	60	60
	BG-550-100-60	550	100	60
	BG-550-120-60	550	120	60
	BG-550-140-60	550	140	60
	BG-640-80-10	640	80	10
	BG-640-80-30	640	80	30
	BG-640-80-120	640	80	120
	BG-640-80-240	640	80	240
	BG-640-80-360	640	80	360

Table S2. Summaries of the kinetic parameters for menthol adsorption on BL-25-25-720 (normal-temperature loading) and BG-640-80 (alcohol-thermal loading) fitted using PFO, PSO, and IPD models, respectively.

Kinetic model	Parameter	BL-25-25-720 (normal-temperature preparation)	BG-640-80 (alcohol-thermal preparation)
PFO	q_e	1113.22	5186.9
	K_1	0.692	5.6564
	R^2	0.9221	0.8930
PSO	q_e	1271.98	5381.7
	K_2	0.0007	0.0022
	R^2	0.9368	0.8829
IPD	K_i	327.26	1524.4
	C_i	201.36	2383.1
	R^2	0.8643	0.4234

Table S3. Summaries of the kinetic parameters for menthol release from normal-temperature- and alcohol-thermal-loaded BMMs obtained by fitting the first-order, Higuchi, and K–P models, respectively.

model	parameters	BL-10 -25-720	BL-15 -25-720	BG-280 -80-60	BG-680 -80-60	BG-550 -60-60	BG-550 -80-60	BG-550 -100-60	BG-640 -80-30	BG-640 -80-120
first-order	K_1	0.722	1.437	0.8189	1.4645	1.1887	1.0391	2.7779	1.14	1.2649
	a	0.007	0.005	0.0577	0.1407	0.1644	0.1717	0.1355	0.1385	0.1117
	R^2	0.9517	0.8282	0.9187	0.9871	0.9889	0.9923	0.9817	0.9943	0.9968
Higuchi	K_H	0.002	0.0013	0.0212	0.0466	0.0583	0.0622	0.0393	0.0494	0.039
	c	0.0015	0.0018	0.009	0.0413	0.0377	0.0331	0.0592	0.0302	0.0273
	R^2	0.8923	0.7956	0.9553	0.7378	0.8440	0.8605	0.6402	0.8280	0.7956
K–P	K_{KP}	0.004	−15.00	0.0328	0.1027	0.1112	0.1108	0.1142	0.0929	0.0777
	n	0.2804	-5×10^{-5}	0.3278	0.2162	0.2590	0.2815	0.137	0.2642	0.2467
	c	-1×10^{-4}	15.00	-4×10^{-5}	−0.0032	−0.0039	−0.0051	−0.0007	−0.004	−0.0032
	R^2	0.9649	0.9609	0.9949	0.9061	0.9519	0.9446	0.9693	0.9308	0.9203
